# Assessing the Delivery of Coordinated Care to Patients with Advanced Chronic Kidney Disease in Ontario, Canada: A Survey of Patients and Healthcare Professionals

**DOI:** 10.5334/ijic.5587

**Published:** 2021-06-22

**Authors:** Jenna M. Evans, Sarah M. Wheeler, Saurabh Sati, Sharon Gradin, Marnie MacKinnon, Peter G. Blake

**Affiliations:** 1 DeGroote School of Business, McMaster University, Hamilton, ON, Canada; 2 Ontario Health (Ontario Renal Network), Toronto, ON, Canada; 3 London Health Sciences Centre, London, ON, Canada

**Keywords:** integrated care, coordinated care, continuity of care, multi-disciplinary, inter-professional, inter-organizational, chronic kidney disease, nephrology

## Abstract

**Introduction:**

Patients with advanced Chronic Kidney Disease (CKD) have complex health needs, and thus require care that is coordinated across professionals and organizations. This study aimed to describe the extent of coordinated care delivery for patients with advanced CKD from the perspectives of both patients and healthcare professionals.

**Methods:**

The Coordination Scale of the Patient Assessment of Chronic Illness Care (PACIC-26) survey was administered to a random sample of 14,257 patients on maintenance dialysis or receiving care in end-stage kidney disease preparation clinics in Ontario, Canada. A five-item survey was administered to 596 multidisciplinary nephrology professionals.

**Results:**

Among the 1,925 patient respondents, 67% reported they had been referred to an allied health professional; 19% had been encouraged to attend programs in the community; and 34% had been told how their visits with other types of doctors helped their treatment (% reporting “always” or “most of the time”). Patient responses were significantly different by treatment modality/setting, but not by gender or geographic location of treatment facility. Among the 276 professional respondents, 37% reported their patients’ care was well-coordinated across settings; 56% reported participating in interdisciplinary care planning discussions; and 53% reported they are aware of appropriate home and community services to support their patients (% reporting “always” or “most of the time”).

**Conclusion:**

The results suggest that care for patients with advanced CKD in Ontario is not consistently coordinated. Healthcare professionals may enhance patient perceptions of coordinated care through explicit communication with patients about how the professionals they see and treatments or services they receive influence their overall health and well-being. At a systems level, there is a need to improve professional awareness of and linkages to home- and community-based services.

## Background

Patients with advanced Chronic Kidney Disease (CKD) receive care from multiple professionals within and across an array of clinical settings [1]. The reason their care frequently crosses professional and organizational boundaries is two-fold. First, given the nature of the disease, the continuum of advanced CKD care is complex and involves primary care, specialist nephrology care, dialysis, transplantation and palliative care [2,3,4,5,6]. Transitions along this continuum are often fragmented. For example, referral from primary care to a nephrologist often occurs late in the course of the disease [7], dialysis and transplant patients may be treated by different teams, even within the same clinical setting [2], and those who develop end-stage kidney disease (ESKD) infrequently access palliative care services, such as advanced care planning [3,8]. Second, in patients with CKD, multimorbidity – the presence of two or more chronic medical conditions – is frequent [9,10,11]. For example, many of these patients also have diabetes and heart disease and often receive care from multiple specialists, in separate clinical settings, each with distinct clinical practice guidelines. They may also regularly visit their primary care physician and receive home- and community-based services. The home and community services sector delivers a wide range of services, many of which support people living with complex care needs. Examples include nutrition programs, transportation services, respite and adult day programs, mental health and addictions services, supportive housing programs, and home care services (e.g., nursing, occupational therapy, personal support).

Insufficient attention to transitions along the continuum of CKD care and to the concurrent management of comorbid conditions can contribute to high treatment burden for patients and their families, poor clinical outcomes, and high health system costs [1,11,12,13]. To optimize their experiences and outcomes, these patients require care that is *coordinated* over time and across professional and organizational boundaries [1,2,9,10,11]. Coordinated care is defined as the deliberate organization of patient care activities between two or more providers involved in a patient’s care [14]. The domain of “care coordination” can be divided into three dimensions: (1) care coordinated *within* a care team, (2) care coordinated *across* care teams, and (3) care coordinated between care teams and home and community resources [15].

Research on coordinated care for patients with CKD focuses primarily on the co-location of multidisciplinary professionals in hospital-based CKD clinics, including nurses, nephrologists, dietitians, social workers, and pharmacists. This type of care coordination can be classified as “coordination within a care team” [15]. Studies of multidisciplinary care for patients with CKD have produced conflicting results, but recent meta-analyses demonstrate positive outcomes including slower declines in eGFR, reduced need for temporary hemodialysis catheters, fewer hospitalizations, decreased all-cause mortality, and enhanced blood pressure control [16,17,18]. These positive effects were more pronounced in patients with severe CKD (stages 4 and 5) receiving care from a *diverse* multidisciplinary team [16]. Such care can also be more cost-effective [19,20].

The literature on coordinated care for patients with CKD also emphasizes the interface between primary care and nephrology, which can be classified as “coordination across care teams” [15]. This body of work largely consists of studies of the extent and consequences of late referral [7] and of enablers and barriers to collaboration [21,22,23]. Few studies test coordinated care interventions aimed explicitly at bridging primary care and nephrology via shared care models that go beyond the use of one-way consult letters or notes [24,25,26].

Despite advances in our understanding of multidisciplinary CKD care and the primary care-nephrology interface, it remains unclear to what extent patients with advanced CKD receive coordinated care across multiple dimensions of the construct – within their CKD care team, across care teams, and between care teams and home and community resources [15]. It is also unclear how patients’ experiences of coordinated care may vary based on socio-demographic and clinical factors [17]. Best practice dictates coordinated care is best measured using reports from patients and healthcare professionals themselves [15]. Only patients can speak to the extent to which they *experienced* coordinated care and only professionals can speak to the extent to which they observed and/or delivered coordinated care throughout a patient’s disease trajectory. Yet, the perspectives of both patients and healthcare professionals are rarely used to assess coordinated care delivery for patients with CKD [28].

The aim of this study was to describe the perceived level of coordinated care for patients with advanced CKD in the Canadian province of Ontario across multiple dimensions of the construct and from the perspectives of both patients and multidisciplinary healthcare professionals. We also examined differences in perceptions of coordinated care based on patient gender, treatment modality/setting, and geographic location of treatment facility.

## Methods

### Context

Over 12,000 people in Ontario, Canada are being treated with maintenance dialysis [29]. One quarter of them manage their dialysis treatment in their own home [29]. In addition, over 10,000 Ontarians with advanced CKD receive care from provincially funded multidisciplinary care kidney clinics (MCKCs) [29]. MCKCs are hospital-based clinics of co-located multidisciplinary professionals, typically including a nephrologist, nurse, pharmacist, dietitian, and social worker. MCKCs target patients with advanced CKD who are at high risk of progressing towards ESKD. To be eligible for these clinics, patients must have either CKD stage 5 or a probability greater than 10% of requiring renal replacement therapy within 2 years based on the Kidney Failure Risk Equation [30].

The Ontario Renal Network (ORN), a unit of Ontario Health, funds and oversees the delivery of CKD care through 27 Regional Renal Programs (RRP) [31]. Each RRP consists of one hub hospital and many have satellite sites located within other hospitals or community settings.

### Survey Instruments

To measure patient perceptions of coordinated care, we used the Coordination Scale of the Patient Assessment of Chronic Illness Care (PACIC-26) Survey (Supplementary Materials). PACIC-26 is based on the Chronic Care Model and assesses patient perceptions of care in five areas, one of which is coordinated care [32]. PACIC-26 has sound psychometric properties and the Coordination Scale, specifically, has high internal reliability [33]. The survey items in the Coordination Scale map on to the three core dimensions of coordinated care [15]. We dropped one item from the Coordination scale (the item on follow-up) because it measures continuity of care rather than coordination of care [34] and had the lowest standardized factor loading, item reliability, and item fit, all of which fall below established standards [32].

To measure professional perceptions of coordinated care, we developed four survey items (Supplementary Materials) that align with the three core dimensions of coordinated care [15]. The four survey items were pre-tested and revised with two nephrologists, a nurse, a social worker, and a person with CKD. We added one open-ended question on barriers to coordinated care delivery. These survey items were included as a sub-section at the end of a broader survey measuring professional practice and perceptions of palliative care delivery to people with CKD. At the beginning of the sub-section, survey respondents were explicitly asked to shift their thinking away from palliative care to the full range of services and professionals that their patients may utilize or benefit from.

### Data Collection

The patient survey was mailed by the National Research Corporation Health (NRC Health) during Summer and Fall 2017 to a random sample of 14,257 patients with advanced CKD who were on dialysis or attending a MCKC. Patients had the option of completing the survey on paper, online, or by phone.

The survey of nephrology professionals employed by the RRPs was administered by the ORN during Fall 2017 via the Survey Monkey online platform to 597 professionals across Ontario. Professional groups most likely to have knowledge of or be involved with care coordination were jointly identified by the research team and ORN leaders and included nephrologists, nurse practitioners, MCKC nurses, in-center hemodialysis nurses, home dialysis nurses, social workers, and program administrators. The ORN requested contact information for all professionals in these roles from all RRPs in the province, and a link to the survey was e-mailed to these individuals.

### Data Analysis

Respondents who were patients were removed from the dataset if they did not answer at least 50% of the care coordination survey items. For the patient survey analysis, the patient groups were combined into the following categories: (1) “Home”, which included patients receiving hemodialysis or peritoneal dialysis at home, (2) “In-Center”, which comprised patients receiving hemodialysis in-center, and (3) MCKC. To compare patient responses by geographic location of treatment facility we used Statistics Canada’s Population Center and Rural Area Classification [35]: Rural area, small population center (1,000–29,000), medium population center (30,000–99,999), and large urban population center (100,000 or greater). For the healthcare professional survey analysis, respondents were removed from the analysis if they did not complete the demographic questions or at least 50% of the survey items. We grouped nephrology program managers, coordinators, and other administrative staff together under “Administration”. For both surveys, analyses were conducted by assigning the following numbers to each response option: 1-Never, Almost Never, Strongly Disagree, 2-Generally Not, Disagree, 3-Sometimes, Neither Disagree nor Agree, 4-Most of the Time, Agree, 5-Always, Almost Always, Strongly Agree.

We performed chi-squared tests to assess demographic differences between respondents and non-respondents, multi-variate analyses of variance across all survey items to assess overall between group differences, and uni-variate analyses of variance and post-hoc t-tests to look for between group differences for each survey item individually. Partial eta-squared was used to determine magnitude of effects. Group comparisons were performed between genders, treatment modalities/settings, and geographic locations of treatment facilities for the patients, and between professional roles for the providers. Missing data in the retained sample was removed in pair-wise fashion for each analysis. All quantitative analyses were conducted using IBM SPSS Statistics Version 25. Responses from healthcare professionals to the open-ended question on barriers to coordinated care delivery were organized into categories and recurrent themes were noted.

## Results

### Patient Survey Results

The patient survey garnered a 17% response rate (n = 2,447) and 1,925 patients were ultimately eligible for inclusion in the analysis having answered sufficient items pertinent to these analyses. A comparison of respondents and non-respondents revealed no differences in response rate between genders and geographic locations of treatment facilities, however a difference in distribution across treatment modalities/settings was observed. Patients receiving home dialysis were more likely to respond to the survey than patients receiving in-center treatment [X^2^ (2, N = 14255) = 46.203, p < .001].

Almost half of patients included in the analysis were on maintenance dialysis and the rest attended MCKCs. ***Table 1*** summarizes their demographic characteristics. There were no significant differences in patient responses by gender or geographic location of treatment facility.

**Table 1:** Patient Demographic Information (n = 1,925).


DEMOGRAPHIC CHARACTERISTIC	N (%)

*Gender*	

Male	1,155 (60%)

Female	770 (40%)

*Treatment Modality/Setting*	

Multi-Care Kidney Clinic (MCKC)	1,001 (52%)

In-Center Dialysis	616 (32%)

Home Dialysis	308 (16%)

*Geographic Location of Treatment Facility*	

Large Urban Population Center	1,367 (71%)

Medium Population Center	404 (21%)

Small Population Center	106 (5.5%)

Rural Area	48 (2.5%)


**Figure 1:**
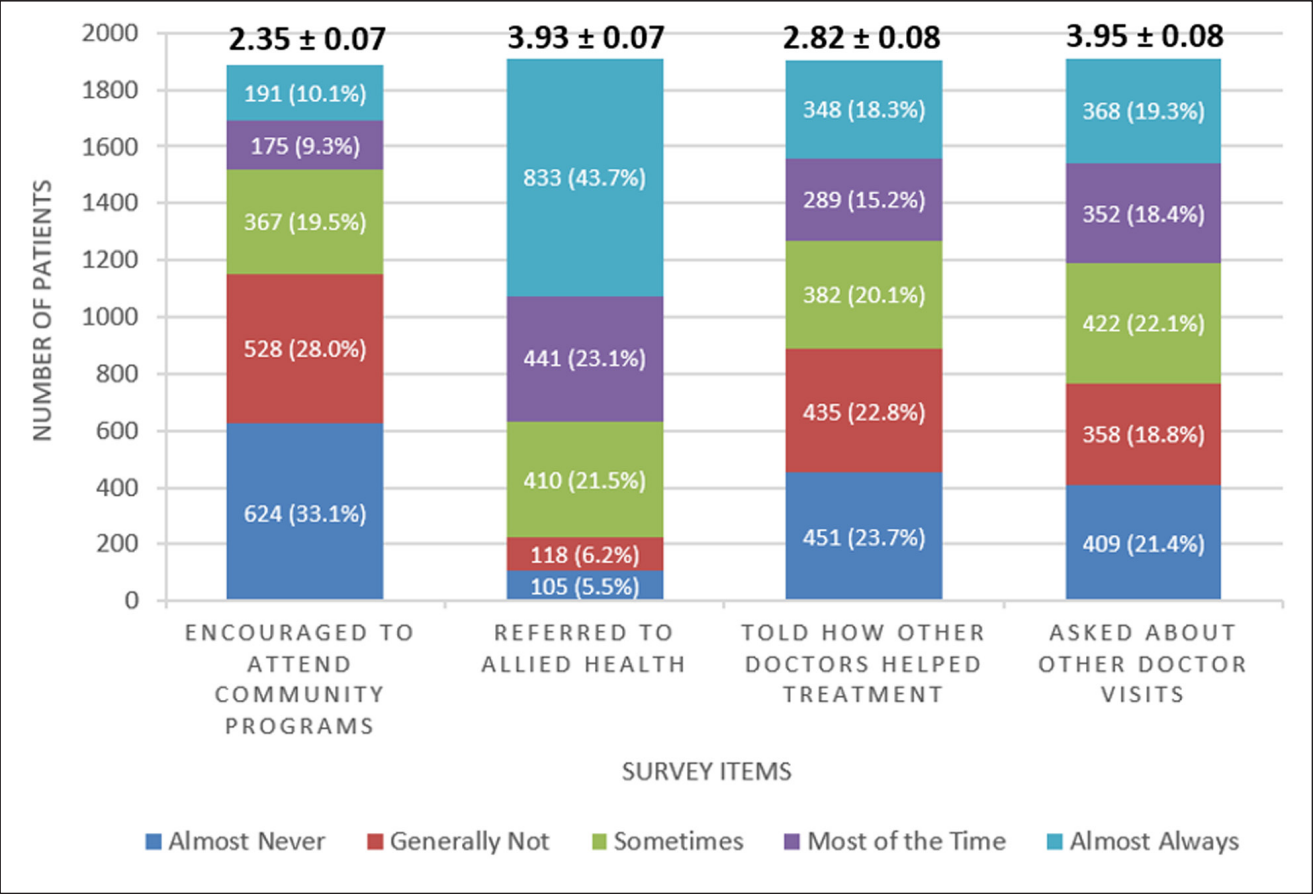
**Summary of Patient Survey Responses (n = 1,925)**. Each stacked bar depicts the number and percentage of respondents per response option per survey item. Above each bar are the mean and standard error of the mean for that survey item.

***Figure 1*** summarizes patient responses to the survey items. Key findings include only 19.4% of patients reported that they were encouraged to attend programs in the community “always” or “most of the time”. A much higher percentage of patients (66.8%) reported that they were referred to an allied health professional, specifically a dietitian, health educator, or counselor. Finally, just over a third of patients reported that they were told how their visits with other types of doctors helped their treatment (33.5%) or were asked how their visits with other doctors were going (37.7%) “always” or “most of the time”.

**Figure 2:**
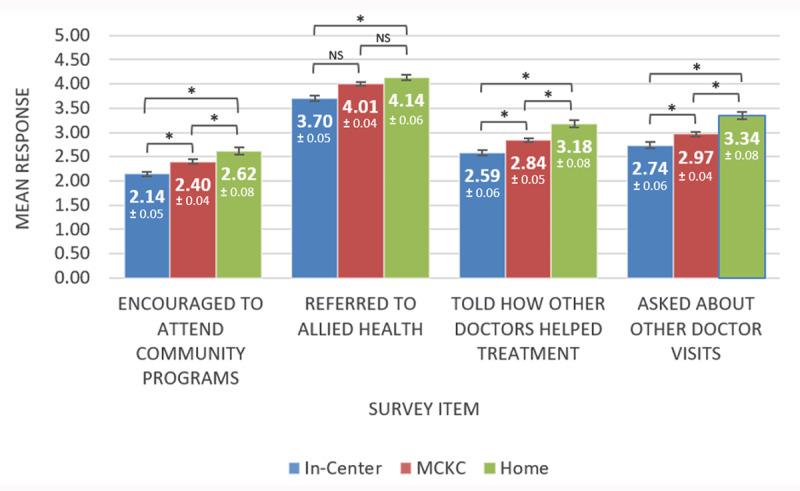
**Patient Survey Responses by Treatment Modality/Setting (n = 1,925)**. Each group of bars depict the mean response per survey item for each of the three treatment modality groups. At the top of each bar are the mean and standard error of the mean (also depicted as an error bar) for each survey item by modality group. A higher mean indicates higher perceived coordination of care. The square brackets indicate that tests of significance were conducted between pairs of groups (i.e., In-Center and Multi-Care Kidney Clinic (MCKC), MCKC and Home, and In-Center and Home). * Significant difference at p < 0.001. ^NS^ Not Significant.

As shown in ***Figure 2***, patient responses were significantly different by treatment modality/setting [F (8,3680) = 8.039, p < .001, η^2^p = .017]. Further analysis revealed that responses were significantly different for all three groups for all four survey items with only one exception. For “referred to allied health”, group differences were not as large. While the overall pattern of results for this item was similar to other items, comparisons between in-center and MCKC and MCKC with home were not significant.

### Healthcare Professional Survey Results

**Figure 3:**
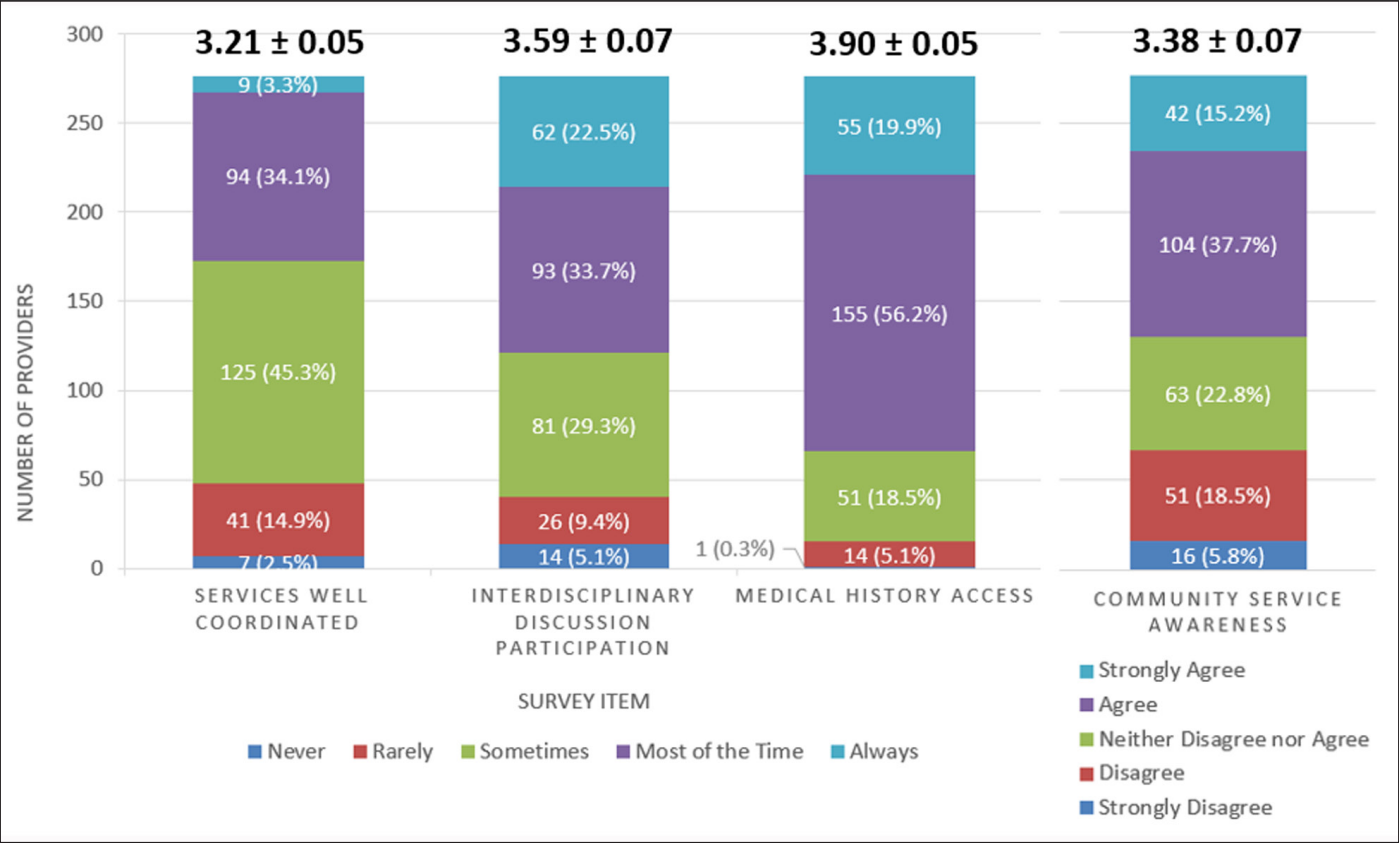
**Summary of Provider Survey Responses (n = 276)**. Each stacked bar depicts the number and percentage of respondents per response option per survey item. Above each bar are the mean and standard error of the mean for each survey item.

The healthcare professional survey garnered a 52% response rate (n = 314) and data from 276 professionals were included in the analysis. ***Table 2*** summarizes their demographic characteristics. ***Figure 3*** summarizes the results of the survey of healthcare professionals. Less than half of healthcare professionals (37.4%) reported their patients’ care was well-coordinated across settings “always” or “most of the time”. Over half of healthcare professionals (56.2%) reported they participate in inter-disciplinary discussions “always” or “most of the time” to develop care plans for their patients, and 52.9% “strongly agreed” or “agreed” that they are aware of appropriate home and community services to support their patients. Most healthcare professionals (76.1%) reported the medical history of their patients was easily accessible “always” or “most of the time”.

**Table 2:** Healthcare Professional Demographic Information (n = 276).


DEMOGRAPHIC CHARACTERISTIC	N (%)

*Professional Role*	

Nurse*	60 (21.7%)

Home dialysis nurse	56 (20.3%)

Nephrologist	53 (19.2%)

Social worker	50 (18.1%)

Administration	39 (14.1%)

Nurse practitioner	18 (6.5%)

*Years in Practice*	

5 years or less	58 (21.0%)

6–10 years	37 (13.4%)

11–20 years	103 (37.3%)

21 years or more	78 (28.3%)

*Age*	

25–34 years old	15 (5.4%)

35–44 years old	63 (22.8%)

45–54 years old	115 (41.7%)

55–64 years old	80 (29%)

65 years and older	(1.1%)


* This category includes both MCKC nurses and in-center hemodialysis nurses. Due to the wording in the survey, we were unable to distinguish between these two groups.

There were no significant differences between professional groups on the extent to which their patients’ care was well-coordinated and accessibility of patients’ medical history. However, nephrologists reported participating in inter-disciplinary discussions more frequently than home dialysis nurses and administrators [F (1,5) = 3.971, p = 0.002, η^2^p = .068], and more social workers were aware of appropriate home and community services than other professional groups [F (1,5) = 15.213, p < 0.001, η^2^p = .220]. These few significant results are not surprising given the nature of their respective professional roles.

Open-ended responses from healthcare professionals (n = 142) revealed four key barriers to coordinated care delivery for patients with CKD: (1) Availability of and access to community-based services, (2) a lack of awareness of community-based services among healthcare professionals, (3) time and effort needed to build required relationships, processes and habits to support coordinated care, and (4) a general lack of resources and capacity to support coordinated care delivery.

## Discussion and Conclusion

The survey results suggest that care for patients with advanced CKD in Ontario is not consistently perceived as coordinated, particularly across care teams that span clinical settings and with home and community services. However, compared with PACIC survey data on coordination from patients with other conditions, our patient sample generated a notably higher average of 3.0 versus 1.9 – 2.7 in other studies [33,36]. Yet, we included 4 out of 5 survey items from the Coordination Scale of the PACIC survey. Nevertheless, when we include the 5^th^ survey item in our calculation, the overall average score is 2.9, still considerably higher than most scores reported in other studies. This higher score may be due to the nature of nephrology – care is delivered to a relatively small number of patients with complex medical, dietary, and social needs and very high attendance rates in hospital-type facilities.

Treatment modality/setting seemed to influence perceptions of coordinated care, though the effect size was very small. Patients on in-center hemodialysis reported the lowest levels of coordinated care, and thus might benefit most from efforts to coordinate care [37]. Those in MCKCs fared better while patients on home dialysis reported the highest levels of coordinated care. Some caveats are appropriate here. Patients on in-center hemodialysis are on average older and frailer and may have more co-morbidities and cognitive impairments than those on home dialysis [38,39]. They may be less aware of home- and community-based services and/or may take a less active role in their care compared with those on home dialysis. Given the small effect size, further research is warranted on whether and how patient perceptions of coordinated care may vary by treatment modality/setting.

Some results may be explained by how care is funded and delivered in Ontario. For example, a much higher percentage of patients reported they were referred to an allied health professional compared with those that reported they were encouraged to attend programs in the community. This discrepancy may be explained by the fact that funding for hospital-based dialysis programs (including MCKCs) requires care from allied health professionals, which may facilitate those linkages in comparison with linkages to external community-based programs and professionals.

The results highlight two key opportunities for strengthening coordinated care delivery. First, there is a need to improve professional awareness of and linkages to home- and community-based services. Only about half of healthcare professionals reported that they are aware of appropriate home and community services to support their patients and less than 20% of patients said that they were encouraged to attend programs in the community. Second, just over a third of patients reported being told how their visits with other types of doctors helped their treatment or being asked how their visits with other doctors were going. These results suggest that healthcare professionals can enhance patient perceptions of coordinated care through explicit communication with patients about how the professionals they see and treatments or services they receive influence their overall health and well-being.

Although this study was focused on patients with CKD, there are general implications for the field of coordinated care. First, there is often a reliance on proxy indicators for coordinated care, such as readmission rates. Although surveys have limitations, their strength is that they explicitly measure views and experiences of coordinated care at a clinical level and can complement or extend other types of data. Second, there is potential to enhance our understanding of coordinated care delivery by collecting data on perceived levels of coordinated care from *both* patients and healthcare professionals. A review of 305 survey instruments measuring aspects of coordinated care found that the majority were administered to patients (60%, n = 228) with far fewer administered to healthcare professionals (20%, n = 77) [40]. In a review of coordinated renal care interventions, the opposite was found: patient views were rarely measured (6%, n = 2) [27]. In our study, collecting both patient and professional views enabled a more robust assessment of perceived levels of care coordination.

This study has limitations, some of which provide direction for future research. First, care coordination is a complex multi-dimensional construct. Yet, we assessed coordinated care delivery using only 4 survey items each for patients and healthcare professionals, respectively. Although our items collectively addressed the three core dimensions of coordinated care [15], individual survey items are unlikely to capture the nuance of each dimension. Second, the healthcare professional survey did not distinguish between those working in a dialysis unit only, MCKC only, or both. Only nurses working in the home setting were distinguishable. Hence, we were unable to conduct sub-analyses based on professional placement in the CKD continuum of care. Third, despite a large patient sample size (n = 1,925) the patient response rate (17%) suggests the results may be biased. However, low response rates are not unusual in surveys of patients, particularly with older, frailer populations who may be over-surveyed [41]. Fourth, data on patient age and comorbidities (such as diabetes), which may have helped explain the results, were not included in the NRC disclosure to the ORN. We were also unable to assess whether the mode of survey completion by patients – paper, online, or phone – influenced results because this information was not included in the NRC disclosure. However, analyses in other studies using the PACIC-26 survey have found no differences in scores among respondents using varied modes of completion [33]. Fifth, the results may not be generalizable to other healthcare systems given differences in the organization and management of CKD care. Finally, the study is subject to limitations associated with survey methodology, including individual differences in interpreting and responding to questions, recall bias, and nonresponse bias. For example, some patients may have interpreted “attending community programs” as referring only to programs they physically go to even though home care and palliative care visits to their residences are also “community programs”. Furthermore, the results from the two surveys were not directly comparable due to minor differences in language of the survey items and scales.

Future research should use validated surveys, such as the Rainbow Model of Integrated Care Measurement Tool, to comprehensively measure the constructs of coordinated and integrated care from the perspectives of both patients and healthcare professionals [42]. Designing surveys for patients and professionals that measure the same constructs using *identical* language may allow for more direct comparisons of patient and professional views. Future studies should also examine the influence of patient-provider communication on patient experiences of coordinated care. Both the literature and the open-ended comments from healthcare professionals in our survey emphasize system-level barriers to care coordination which are often challenging to address and require policy changes. However, the survey results suggest that we may impact patient perceptions of coordinated care through smaller-scale changes to patient-provider communication, which is within the control of individual professionals and leaders. Standardized measurement of coordinated care delivery over time using surveys can support local quality improvement and broader system transformation towards more consistent coordinated care delivery for patients with CKD.

## Data Accessibility Statement

Requests by qualified researchers to access our de-identified raw data may be made directly to the authors and will be subject to review and approval by Ontario Health (Ontario Renal Network).

## Supplementary Materials

### Patient Survey: Coordination Scale of PACIC-26

Over the past 6 months, when I received care for my kidneys, I was:

1. Encouraged to attend programs in the community that could help me
  • Response options: Almost Never, Generally Not, Sometimes, Most of the Time, Almost Always
  • Dimension of coordinated care: Coordination with home and community resources
2. Referred to a dietitian, health educator, or counselor
  • Response options: Almost Never, Generally Not, Sometimes, Most of the Time, Almost Always
  • Dimension of coordinated care: Coordination within care team
3. Told how my visits with other types of doctors, like the eye doctor or surgeon, helped my treatment
  • Response options: Almost Never, Generally Not, Sometimes, Most of the Time, Almost Always
  • Dimension of coordinated care: Coordination across care teams
4. Asked how my visits with other doctors were going
  • Response options: Almost Never, Generally Not, Sometimes, Most of the Time, Almost Always
  • Dimension of coordinated care: Coordination across care teams

### Healthcare Professional Survey

1. How often were the services for each of your chronic dialysis and/or MCKC patients well-coordinated across primary, specialist nephrology (including transplantation), and palliative care in hospitals and in the community? *Services are well-coordinated when providers share important clinical information, have clear shared expectations about their roles, and ensure effective referrals and transitions take place*.
  • Response options: Never, Rarely, Sometimes, Most of the Time, Always
  • Dimension of coordinated care: Coordination across care teams
2. How often did you participate in interdisciplinary discussions to develop individual care plans for your chronic dialysis and/or MCKC patients? *Interdisciplinary discussions include a range of healthcare professionals, such as nephrologists, nurse practitioners, nurses, pharmacists, social workers, dietitians, dialysis technicians, community partners and primary care providers*.
  • Response options: Never, Rarely, Sometimes, Most of the Time, Always
  • Dimension of coordinated care: Coordination within care team AND Coordination across care teams
3. How often was the medical history of your chronic dialysis and/or MCKC patients easily accessible? *Medical history is a record of information about a person’s health, which may include information such as test results, illnesses, surgeries, medications taken and health habits*.
  • Response options: Never, Rarely, Sometimes, Most of the Time, Always
  • Dimension of coordinated care: Coordination within care team, Coordination across care teams, and coordination with home and community resources
4. To what extent do you agree or disagree with the following statement: “I am aware of appropriate home and community services to support my chronic dialysis and/or MCKC patients”? *Examples include home nursing care, transportation services, hospice services, supportive housing programs, adult day programs and mental health and addictions services arranged through your local LHIN Home and Community Care Division (formerly CCAC) or privately*.
  • Response options: Strongly Disagree, Disagree, Neither Disagree nor Agree, Agree, Strongly Agree
  • Dimension of integrated care: Coordination with home and community resources
5. Please comment on key barriers to integrated care delivery for chronic dialysis and/or MCKC patients.
